# Non-Motor symptoms in Portuguese Parkinson’s Disease patients: correlation and impact on Quality of Life and Activities of Daily Living

**DOI:** 10.1038/srep32267

**Published:** 2016-08-30

**Authors:** Paulo Bugalho, Tânia Lampreia, Rita Miguel, Marcelo D. Mendonça, André Caetano, Raquel Barbosa

**Affiliations:** 1 Department of Neurology, Hospital de Egas Moniz, Centro Hospitalar de Lisboa Ocidental, Rua da Junqueira, 126 1349-019 Lisboa, Portugal; 2 CEDOC, Chronic Diseases Research Center, NOVA Medical School Campo Mártires da Pátria 130, 1169-056 Lisboa, Portugal

## Abstract

The prevalence of non-motor symptoms (NMS) in Parkinson’s Disease (PD) has varied between studies. Their interrelation isn’t totally understood. Also, the relative importance of each symptom, regarding its impact on activities of daily living (ADL) and health related quality of life (HRQL), remains debatable. We assessed all PD patients attending a Portuguese tertiary movement disorders center during one year (n = 134), with ADL, HRQL and other clinical scales approved for identifying the most relevant NMS in PD. All patients had at least one NMS. Sleep/fatigue, affect/cognition, attention/memory were the most frequent complaints, and their prevalence, above 80%, was higher than in most studies. There were significantly correlations between: sleepiness, psychosis and cognition; gastrointestinal, cardiovascular symptoms and pain; depression and apathy; anxiety and insomnia; olfaction, weight and hyperhidrosis. Depression/apathy exerted the strongest influence on HRQL and non-tremor motor dysfunction on ADL. Compared to studies in other countries, we found a higher prevalence of NMS, which could be specific of this population. The interrelation between NMS could be related to degeneration of different brain structures. NMS exert a stronger influence than MS in HRQL, which should be taken in account regarding treatment options.

Although Parkinson’s Disease (PD) is classified as a movement disorder, non-motor symptoms (NMS) also occur^1^,^2^,^3^. Several studies have assessed the relative prevalence of non-motor symptoms, but results have been discordant, and have varied between different world regions, depending on particular epidemiological features. Cognitive dysfunction, for instance, was found to be increased in populations with lower education levels^4^, the total prevalence of NMS was found to be increased in regions with less health care availability^5^ and the prevalence of hallucinations lower in countries with less access to dopaminergic treatment^4^. The relation between symptoms has also been assessed, suggesting a relation between cognitive deficits and non-tremor motor symptoms^6^; depression and cognitive dysfunction^7^; psychosis and sleep dysfunction^8^,^9^; apathy and depression or executive dysfunction^10^. However, many studies have focused only on a particular set of symptoms. Symptom aggregation pattern, which can be informative for evaluating the underlying pathophysiology of the complaints, has seldom been evaluated with principal component analysis methods.

It has been reported that NMS could contribute to the incapacity and influence health related quality of life (HRQL)^11^,^12^,^13^,^14^,^15^, but it is still unclear what the relative weight of each symptom is. Furthermore, there is evidence that health preferences vary between countries as does the perception of self-rated health quality^16^. In Portugal, there are few studies evaluating the impact of neurologic disorders in HRQL and there is none, as far as we could ascertain, that has studied the prevalence and impact of different symptoms in PD. Being a region with low income and lower education levels (particularly regarding the more aged segments), compared to other European countries^17^, it can be hypothesized that this assessment could differ from studies performed in other countries.

The objectives of the present study were therefore to evaluate a Portuguese tertiary care population of PD patients, with a wide set of instruments and scales for assessing NMS, in order to determine:

- The prevalence of non-motor symptoms.
- The correlation between the different motor and non-motor symptoms.
- The impact of motor and non-motor symptoms on HRQL and Activities of Daily Living (ADL).

## Results

One hundred and forty three patients attended consultation at Egas Moniz Hospital outpatient clinic for movement disorders during the studies’ one year period. Nine patients were excluded from analysis: 4 refused assessment, 1 had concomitant stroke related deficits (right side pyramidal deficits) and 4 were unable to complete more than 90 percent of the assessment (one had incapacitating knee disease, preventing ambulation, one was recovering from pneumonia and too weak to perform the tests, two had advanced dementia). There were no significant differences between these patients and the ones that were included in terms of age, age of onset, disease duration and gender proportion.

Table 1 shows the samples’ descriptive data. Twenty two patients (15.4%) presented criteria for dementia. Thirty one patients (21.7%) had mild cognitive impairment. Nine patients were under antipsychotics (either quetiapine or clozapine).

**Figure 1:**
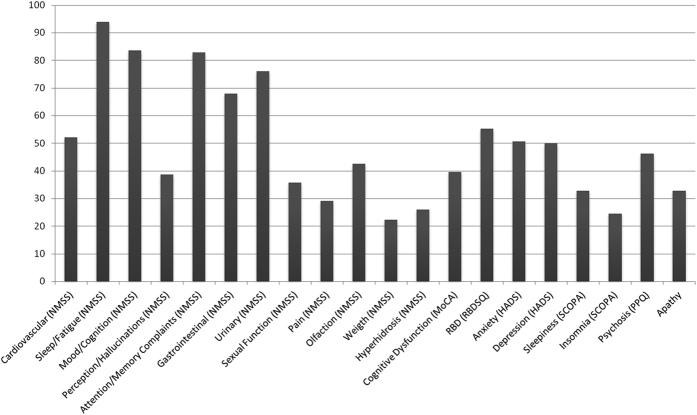
Prevalence of non-motor symptoms in PD.

All patients had at least one NMS in NMSS and 97.7% presented more than one symptom. The mean number of NMS per patient was 6.6 (range 1–12, standard deviation 2.3). Figure 1 shows the relative prevalence of NMS.

Bars represent percentage of patients with non-motor symptoms (from a total of 134), as measured by the Non-Motor Symptom Scale (NMSS), MoCA (Montreal Cognitive Assessment scale) REM Sleep Behavioral Disorder Symptom Questionnaire (RBDSQ); Hospital Anxiety and Depression Scale (HADS), Scale for Outcomes in Parkinson’s Disease (SCOPA), Parkinson’s Psychosis Questionnaire (PPQ) and Apathy Scale.

According to NMSS, sleep/fatigue complaints were the most frequent symptoms, affecting almost all patients, followed by mood/cognition complaints, attention/memory complaints and urinary complaints, all affecting over 70% of the patients. According to the other scales, depression, anxiety, RBD and psychosis were the most frequent symptoms (present in about half of the patients).

Principal component analysis yielded 8 rotated factors, which represented 65.7% of the total sample variance (Table 2. shows loadings of motor and non-motor symptoms on to individual factors). Factor 1 was associated with Dysarthria, Rigidity, Bradykinesia and Gait/Postural Disturbance. Factor 2 with SCOPA daytime complaints, NMSS Perception/Hallucinations score, PPQ score and NMSS Attention/Memory score. Factor 3 was correlated with NMSS gastrointestinal symptoms, NMSS cardiovascular symptoms and NMSS pain. Factor 4 was correlated with HADS Depression scores, Apathy and NMSS Mood/Cognition scores. Factor 5 with HADS Anxiety scores, SCOPA nighttime complaints and NMSS sleep/fatigue scores. Factor 6 was correlated with NMSS olfaction, NMSS weight and NMSS hyperhidrosis scores. Factor 7 correlated with MoCA score, NMSS urinary symptoms and NMSS sexual dysfunction symptoms scores. Factor 8 with RBDSQ score and Tremor.

Table 3 presents the results from the regression analysis performed to evaluate the influence of the different factors on HRQL and ADL. Factor 4 (associated with depression and apathy) exerted the strongest influence on HRQL, both regarding EQ-Index and EQ-VAS scores. It was followed by Factor 1 (non-tremor motor dysfunction) and Factor 5 (insomnia and anxiety). Factor 2 (cardiovascular, gastrointestinal and pain symptoms) was significantly associated with lower values on the EQ-VAS. The strongest influence on ADL (as measured both by Schwab and England and UPDRS II) was exerted by non-tremor motor dysfunction, followed, in decreasing order, by sleepiness/psychosis, cardiovascular/gastrointestinal/pain symptoms and depression/apathy. Factor 5 (insomnia/anxiety) and Factor 7 (MoCA score/urinary and sexual dysfunction) also exerted a significant, but weaker influence on ADL, and only regarding the UPDRS II score.

## Discussion

Our study reveals a high global NMS burden, with all patients presenting at least one NMS and almost every patient presenting more than one symptom. The relative NMS prevalence, as assessed by NMSS, agrees with several other studies^1^,^2^,^3^,^12^ showing sleep/fatigue, mood/cognition, memory/attention and urinary as the most prevalent symptoms. However, the absolute prevalence of the most frequent symptoms, as assessed by the NMSS, is higher than in most studies that used this same scale^2^,^12^, in which percentages above 60 are rare, while in our sample sleep disturbances reached almost 100%, and affect/cognition reached around 80%. This could be caused by cultural, geographic or pattern of health care particularities in our sample, and deserves further investigation, in other Portuguese cohorts. Global population background should be taken in account, as some of these symptoms are highly prevalent in old age population. In particular, we should notice that the Portuguese are an aged population, with reduced health spending costs, when compared with countries where other studies were performed^17^, which increases morbidity in general. Global sleep satisfaction is lower than in other countries^18^ and anxiety and depression symptoms present one of the highest prevalences in European countries^19^. The prevalence of clinical significant symptoms (assessed by scales other than NMSS) was lower, and similar to previous studies^14^, which is accordance with this hypothesis. The relative prevalence of symptoms also differs between the two types of assessment, the main difference being the change in nighttime sleep symptoms frequency, which appears in fifth place, after anxiety, depression, psychosis and cognitive dysfunction. This could be explained by scale related differences –contrary to SCOPA, NMSS includes fatigue related items, which have been reported as highly prevalent in PD patients^2^,^12^. Psychosis prevalence was similar for NMSS and PPQ scales, and higher than in most studies. This was also detected in a previous investigation in early stage Portuguese PD patients, and could be related to the older mean age of our PD population^7^ or to the tendency to tolerate low intensity psychotic symptoms rather than using neuroleptics, which is suggested by the reduced number of patients who were under antipsychotics compared to those who scored positive in the psychosis scales.

Principal component analysis suggested different patterns of connection between the symptoms. There was a dissociation between tremor and non-tremor MS, substantiating a different pathophysiological background. NMSS cognitive complaints did not co-varied with objective cognitive deficits measured by MoCA. This suggests a dissociation between subjective memory complains (SMC) and objective cognitive dysfunction. It could be due, however, to differences in the cognitive capacity which is being measured by the scales – NMSS evaluates memory, while MoCA has a higher load on executive functions. Our results show, on the other hand, a significant relation between cognitive complaints and sleepiness. This could be explained by the effects of sleepiness episodes on daily cognitive activities that require alertness. Because sleepiness tends to fluctuate, its effects could be more apparent regarding global impression of cognitive function, as given by the NMSS, than regarding the results of short interval objective examination provided by MoCA, during which voluntary effort could overcome sleepiness. The effect of sleepiness in self-reported cognitive dysfunction and the notion that daytime sleep complaints are a separate phenomenon from, rather than a consequence of sleep deprivation, corroborate findings of previous studies^20^. The relation between sleepiness and hallucinations supports the hypothesis that hallucinations could represent the intrusion of oneiric imagery in the wake state during sleepiness episodes^10^. Both symptoms are intensified by dopaminergic treatment, which could also contribute to this connection. Cardiovascular and gastrointestinal symptoms share a common origin in autonomic system dysfunction, which could explain their aggregation in a single factor. Pain has a multifactorial origin in PD. Neuronal losses have been observed in Lamina I of the posterior spinal horn, which is involved in the gating of painful stimuli coming from peripheral receptors^21^. Spinal neuron degeneration is also involved in autonomic system symptoms, providing a possible explanation for the relation between cardiovascular, gastrointestinal and pain complaints. Apathy is a common symptom in PD, although its physiopathology is still not completely understood, because it is difficult to isolate from neuropsychiatric domains with similar symptoms, like depression and cognitive dysfunction^10^. In our sample, apathy was related to depression, with which it shares some of the symptoms (e.g. anhedonia), suggesting a common etiology and eventually a common therapeutic venue. Anxiety and depression did not correlate with the same factor. The relation between these two psychiatric dimensions in PD has been controversial. Some studies^22^ have found that anxiety and depression are related to different factors in PD patients, suggesting an alternative physiopathology. Anxiety, on the other hand, was correlated with nigh-time sleep problems. This has also been reported by Ratti *et al*.^23^. It seems reasonable to hypothesize that higher anxiety levels could contribute to insomnia in PD by creating a state of hyperarousal, as has been described in non-PD populations. Our results also support a correlation between weight change, olfaction and hyperhidrosis. Sharma *et al*.^24^ also found a significant relation between olfaction loss and weight loss, in a prospective study. While it may be argued that olfaction and taste loss could cause weight change by diminishing food intake, those authors did not found a relation between energy intake and hyposmia and suggested that olfaction loss could be a pre-clinical marker of a non-motor phenotype that would lead to weight loss as the disease progressed.

The present study suggests that although non-tremor motor dysfunction has a significant impact on HRQL, depression/apathy has the stronger influence. This occurred with both HRQL measures, meaning that it was not biased by the presence of a depression related item on EQ-Index. This is in accordance with studies performed in other countries^1^,^12^, suggesting it could be a generalized, cross-cultural finding, to which attention should be drawn, as affective symptoms are potentially reversible, if appropriately treated. Nighttime sleep disturbances also had a significant correlation with HRQL, as previously reported^1^,^13^. Factor 3 (gastrointestinal, cardiovascular and pain symptoms), had a significant impact on EQ-Index, and a still significant, but weaker impact on EQ-VAS. Although both pain and gastrointestinal symptoms have been reported to influence HRQL in previous studies, in the present case we cannot exclude a scale related bias, driven mainly by the pain component of Factor 3, which is included as a specific item on EQ-Index. On the other hand, non-tremor motor dysfunction exerted the strongest negative influence on ADL, followed by sleepiness/memory complaints/hallucinations. Motor dysfunction was found in several studies to have the strongest influence in incapacity^13^. Sleepiness as also been reported to affect ADL^13^, and hallucinations were found to be a major factor for nursing home placement^25^. The influence of both classes of symptoms in patients’ autonomy can represent a major clinical problem, as dopaminergic drugs, the main treatment for motor symptoms, tends to worsen both hallucinations and sleepiness.

Our study presents some advantages. It is, as far as we know, the only study to evaluate the global prevalence of NMS and their impact in HRQL in a Portuguese population, widening previous findings to a different geographical and cultural setting, and suggesting particularities that could be further investigated in other Portuguese samples. The use of a wide set of scales could also be considered an advantage, as they allowed to assessed the great majority of symptoms described in PD patients. Using different scales for the same domains also permits to diminish scale related biases. As the main drawbacks, we should refer the relative small number of patients (if compared to some of the multicenter studies) and the lack of a control group, which could have allowed us to distinguish between disease and age related differences. Our objective was to assess the impact of the different symptoms in HRQL, and our concern was to use a wide set of scales, so not to exclude important complaints. Other factors, however, like level of exercise, occupation, income level or marital status, are also important determinants of HRQL. Not accounting for these can be considered a drawback of this study. Evaluation was performed during *on* state. This, of course, can underestimate the importance of motor symptoms on HRQL during *off* state. One could also argue that the impact of non-dopaminergic medication could have biased these results, because some symptoms respond better than other to the recommended medication, and differently depending on the disease stage. This, however, was not addressed in our study, which could be considered as a limitation.

In conclusion, our results show a high prevalence of non-motor symptoms in PD patients, insomnia, depression and urinary symptoms being the most frequent. This prevalence was higher than in most investigations, requiring further study in the Portuguese population. The interrelation between symptoms shows an intricate pattern, which could further be assessed in studies that link symptoms with neuropathological changes and brain activation. Our study supports investigations performed in other cultural and geographical contexts, by showing that NMS exert a stronger influence than MS in HRQL, which should be taken in account when planning treatment for PD.

## Methods

### Study design

clinical, cross-sectional, observational, tertiary referral center based study, in a sample of consecutively observed PD patients. Setting and participants: We addressed all consecutive patients fulfilling criteria for PD, who attended Egas Moniz Hospital outpatient clinic for movement disorders, a tertiary referral center in the Lisbon area, Portugal, during the time period between March 2014 and March 2015. We used the UK Brain Bank Diagnostic criteria for PD diagnosis^26^. Exclusion criteria were the presence of significant comorbidities that could interfere with assessment and of signs and symptoms suggesting other causes for parkinsonism (Multiple System Atrophy, Vascular Parkinsonism, Progressive Supranuclear Palsy, iatrogenic parkinsonism). Patients were excluded only if the comorbidities prevented patients from completing the questionnaires or if they represented an extra load of incapacity that couldn’t be accounted by PD alone. The study protocol was applied to all patients except those that refused. Information was collected from caretakers whenever patients were considered unreliable (e.g. patients with dementia, patients without critic regarding their psychotic symptoms) or uninformative (e.g. RBD symptoms). The evaluator decided, by clinical assessment, if the patient was able to rate HRQL autonomously. Less severe cases of dementia were included, if the patient could provide a rational account of his/her choice and this was corroborated by the caretaker (information was considered inadequate if the caretaker reported it to be discordant with the patient reality - e.g. high rates in the mobility section in patients that lacked autonomous ambulation). Severe cases of dementia were excluded from analysis (two cases, as already reported).

Demographic data was collected from patients who refused assessment, if permitted. The study protocol included the collection of demographic data: age at study inclusion, age of disease onset, duration of disease (period, in years, between the first motor symptom experienced by the patient and the date of assessment), education (years of schooling), dopaminergic medication (for patients on dopamine agonist treatment, L-dopa equivalent doses (DED) were calculated, following published data^27^). Assessment took in average 90 minutes and consisted on the application of a battery of tests by movement disorders specialists and trainees, following the order in which they are listed below. All scales are of wide use in Parkinson’s disease, and were chosen according to the Movement Disorders Society recommendations.

### Motor function

Hoehn and Yahr stage^28^, Unified Parkinson’s Disease Rating Scale Part III^29^. Separated scores were derived for tremor, rigidity, bradykinesia, dysarthria and gait/postural stability symptoms, from items 20 and 21, 22, 23 to 27, 18, and 29 to 30, respectively.

### Activities of Daily Living

Unified Parkinson’s Disease Rating Scale Part II^29^ and Schwab and England Activities of Daily Living scale^30^. UPDRS part II consists on a self-evaluation scale, which includes questions regarding the impact of motor dysfunction on speech, swallowing, handwriting, dressing, hygiene, falling, salivating, turning in bed, walking, and cutting food. The Schwab and England Activities of Daily Living scale estimates, in percentage, the patients capacity to deal with activities of daily living, ranging from “completely independent” (100%), to “vegetative functions such as swallowing, bladder and bowel functions are not functioning. Bedridden” (0%).

### Health Related Quality of Life

Euro-Qol (EQ-5D)^31^. The EuroQol measures patient reported HRQL and comprises two sections. The EQ-5D consists of five questions (graded in three levels of severity: 1 = no problem, 2 = moderate problem,3 = severe problem) related to five dimensions of health: mobility, self-care, usual activities, pain/discomfort and anxiety/depression. The EQ-5D generates 243 theoretically possible health states, from which an index score can be derived, according to published recommendations (EQ-Index)^32^. EQ-VAS is a vertical visual analogue scale ranging from 0 (worst imaginable health state) to 100 (best imaginable health state).

### Cognitive Functions

Montreal Cognitive Assessment Scale (MoCA)^33^. The MoCA screening tool is a 30 items scale that evaluates attention, orientation, language, verbal memory, visuospatial and executive functions. It takes approximately 10 minutes to administer. We used age and education related cut-offs for cognitive dysfunction, as provided by the MoCA Portuguese validation study^34^. Patients with cognitive dysfunction and impact in daily living activities (according to the Pill Questionnaire, see below) were classified has having Dementia. Mild Cognitive Impairment was classified as cognitive dysfunction without impact in daily living activities.

### REM Sleep Behavioral Disturbance Symptoms

REM Sleep Behavioral Disturbance Questionnaire (RBDSQ)^35^ - RBDSQ is a screening tool, which proved great sensitivity and reasonable specificity for RBD diagnosis as confirmed by polysomnography. RBDSQ contains 13 questions, covering several aspects of RBD symptom spectrum. We used 6 as the cut-off score for RBD, as proposed by a recent investigation^36^ that validated this scale for use in PD populations.

### Impact of Cognitive Dysfunction on Daily Living Activities

Pill Questionnaire^37^. The Pill Questionnaire was proposed by The Movement Disorder Task Force as a tool to evaluate the impact of cognitive dysfunction in daily living activities, irrespective of motor dysfunction, and consists on questions that assess the patient’ s capacity to manage his/her own medication.

### Anxiety and Depression

Hospital Anxiety and Depression Scale^38^. HADS is a self-assessment instrument comprising 7 depression and 7 anxiety related questions, which are graded in a four point scale (0–3), with a total score which can be derived separately for each dimension by adding each question scores. Scores equal or below seven are considered as non-significant.

### Sleep Disturbance

SCOPA-Sleep^39^ (scales for outcomes in PD) is a self-rating scale created to access sleep quality and daytime sleepiness symptoms in patients with PD, occurring in the previous month. It includes three subscales: a nighttime scale (NS), a single-item quality of sleep scale (which was not used in this study) and a daytime sleepiness scale (DS). The NS contain five items, with four response options that address insomnia related complains (sleep initiation, sleep fragmentation, sleep efficiency, sleep duration, and early wakening). The maximum score of this scale is 15 (indicating worse symptoms), and the suggested cut-off is 6/7. The DS subscale evaluates daytime sleepiness, according to six items, with four response options. Scores range from 0 to 18, and the suggested cut-off score is 4/5.

### Psychosis

Parkinson’s Psychosis Questionnaire^40^: the PPQ is a 14-item screening instrument which includes questions regarding four domains: sleep disturbance, hallucinations and/or illusions, delusions, disorientation. Within a domain, any positive answer triggers inquiries about frequency (1–3 points) and severity (1–3 points). Each subscore is the product of the frequency multiplied by the severity score for that symptom domain. The total score is obtained by summing all subscores. PPQ caseness is defined by at least a positive score on the domains of hallucinations and/or illusions or delusions.

### Non-motor symptoms (global)

Non-Motor Symptom assessment scale for Parkinson’s Disease (NMSS)^3^. The NMSS contains a total of 30 items and evaluates nine non-motor symptoms domains: 1.Cardiovascular symptoms; 2. Sleep/Fatigue; 3. Mood/Cognition; 4. Perception/Hallucinations; 5. Attention/Memory; 6. Gastrointestinal symptoms; 7. Urinary symptoms; 8. Sexual Function symptoms; 9. Miscellanea. The scores for each item are calculated by multiplication of frequency and severity, ranging from 0 (not present) to 12 (maximum frequency and severity) by multiplication of both concepts (‘symptom burden’). The total domain scores are obtained by adding the corresponding item scores. Because Domain 9. represents a miscellanea of different symptoms, these 4 items were considered separately instead of addictively, yielding the following variables: Pain, Olfaction, Weight Change, Hyperhidrosis.

### Apathy

Apathy Scale^10^. This is a 13 item scale, covering several aspects of apathetic behavior. For each questions there are four possible answers: “not at all,” “slightly,” “some,” or “a lot”. (graded from 0 to 3). Total score is the sum of all questions scores, and ranges from 0 to 42, higher scores indicating more severe apathy. A score of 14 or more is considered as clinical significant.

### Data analysis

In order to evaluate the prevalence of each NMS we calculated the number of cases with scores above cut-off in each of the scales. Regarding NMSS, caseness was defined in each domain as at least one positive answer in one of the items (score > 0). Regarding the other scales, caseness was defined by scores above previously published cut-offs values. Although some aspects were evaluated by more than one scale (eg. Sleep Disturbance by SCOPA and NMSS Domain 2), these are not completely overlapping (eg. NMSS Domain 2. assesses both fatigue and nighttime sleep complaints, while SCOPA assesses day time and night time symptoms separately, and does not include fatigue) and in some cases they represent different aspects of the same symptom (e.g. Domain 3. assesses cognitive subjective complaints, while MoCA is an objective marker of cognitive dysfunction). For this reason, we decided to present frequency results from all the scales, which also allows to detect scale related biases. To evaluate the relation between symptoms, we performed a principal component analysis with varimax rotation, in which were included non-motor scores and the five separate UPDRS III scores. Eigenvalues above 1 were considered as loading on one factor. Given the high number of variables, and to avoid collinearity, each patient was given a score on each factor, and these were used in linear regression analysis (see below) instead of the original variables. In order to assess the symptom impact on HRQL and ADL, we performed a linear regression analysis, in which factors were included as independent variables and EQ-5D, EQ-VAS, UPDRS-II and Schwab and England scores as the dependent variable. Chi-Square statistic (or Fisher tests) were used to compare categorical variables. T-test or Mann-Whitney tests (depending on the distribution of the variables) were used to compare continuous variables. p < 0.05 was considered significant.

### Ethics

Patients signed informed consent forms and the study protocol was approved by the ethics committee of Centro Hospitalar de Lisboa Ocidental. Methods were carried out in accordance with the declaration of Helsinki. Regarding patients with dementia, informed consent was obtained from the legal representative.

## Additional Information

**How to cite this article**: Bugalho, P. *et al*. Non-Motor symptoms in Portuguese Parkinson's Disease patients: correlation and impact on Quality of Life and Activities of Daily Living. *Sci. Rep*. **6**, 32267; doi: 10.1038/srep32267 (2016).

## Figures and Tables

**Table 1:** Motor and non-motor symptoms in PD - descriptive data.

	Mean/Frequency	Standard Deviation/percentage	Range
Age	73.3	8.8	42–94
Gender (male)	70	49	
Age of onset	65.9	11.1	28–92
Duration of symptoms	7.6	6.3	1–38
LED	538.4	382.2	0.0–1600.0
Hohen and Yahr stage	2.4	0.9	0–5
UPDRS III Total	28.2	16.8	0–104
UPDRS III Dysarthria	1.13	1.0	0–4
UPDRS III Tremor	4.5	3.6	0–19
UPDRS III Rigidity	4.1	4.0	0–15
UPDRS III Bradykinesia	11.8	7.9	0–36
UPDRS III Gait/Posture	2.5	1.8	0–8
MoCA	18.5	6.8	0–30
RBDSQ	6.32	3.4	1–13
HADS - Anxiety	8.0	4.4	0–20
HADS - Depression	8.3	5.2	0–24
SCOPA-Sleep Daytime	3.4	4.8	0–36
SCOPA-Sleep Nighttime	4.4	3.9	0–15
PPQ	3.8	3.2	0–14
Apathy	11.9	7.7	0–43
NMSS total	60.9	45.8	2–190
NMSS Affection/Cognition	16.4	17.0	0–72
NMSS Perception/Hallucinations	2.0	4.6	0–24
NMSS Sleep/Fatigue	10.0	8.0	0–34
NMSS Cardiovascular	1.5	0.5	1–2
NMSS Attention/Memory Complaints	8.0	9.0	0–36
NMSS Gastrointestinal	4.9	6.4	0–30
NMSS Urinary	7.5	9.1	0–36
NMSS Sexual Function	3.6	6.4	0–24
NMSS Pain	1.5	3.0	0–12
NMSS Olfaction	2.9	4.4	0–16
NMSS Weight Change	1.1	2.7	0–12
NMSS Hyperhidrosis	1.6	3.9	0–24
NMSS Euro-QoL Index	0.3	0.3	−0.6–0.7
NMSS EQ-VAS	62.0	21.2	0–100
UPDRS II	13.9	9.7	0–52
Schwab and England Activities of Daily Living (ADL)	75.5	19.2	10–100

UPDRS - Unified Parkinson’s Disease Rating Scale; NMSS - Non-Motor Symptom Scale; MoCA - Montreal Cognitive Assessment scale; RBDSQ - REM Sleep Behavioral Disorder Symptom Questionnaire; HADS - Hospital Anxiety and Depression Scale, SCOPA - Scale for Outcomes in Parkinson’s Disease; PPQ - Parkinson’s Psychosis Questionnaire.

**Table 2:** Principal component analysis: loadings of motor and non-motor symptoms on individual factors.

	Factor							
	1	2	3	4	5	6	7	8
MoCA	−0.432	−0.349	−0.180	−0.167	0.013	−0.081	0.388	0.313
RBD	0.084	0.389	0.018	0.016	0.287	0.111	0.078	0.606
UPDRS III Dysarthria	0.725	0.293	0.128	−0.004	−0.130	0.075	0.038	0.058
UPDRS III Tremor	0.278	−0.333	0.107	−0.089	−0.189	0.059	−0.015	0.618
UPDRS III Rigidity	0.699	0.157	−0.156	0.224	0.043	0.119	0.072	0.285
UPDRS III Bradykinesia	0.835	0.088	0.105	0.126	0.085	−0.008	−0.077	0.158
UPDRS III Gait/Posture	0.716	0.077	0.266	0.093	0.156	0.015	0.230	−0.146
HADS depression	0.319	0.141	0.237	0.526	0.358	−0.073	−0.002	0.046
HADS anxiety	−0.112	0.042	0.447	0.389	0.504	0.039	−0.074	0.135
SCOPA daytime	0.005	0.592	0.250	−0.129	−0.327	−0.146	0.231	0.220
SCOPA night time	0.082	0.026	0.098	0.069	0.819	0.007	0.047	−0.028
PPQ	0.291	0.737	0.031	0.171	0.168	0.076	−0.114	−0.002
Apathy	0.206	0.137	−0.001	0.785	−0.064	0.188	0.161	−0.129
NMSS Hyperhidrosis	0.069	0.033	0.020	−0.221	0.310	0.620	0.171	−0.161
NMSS weight loss	0.098	0.006	0.182	0.211	−0.067	0.667	0.003	0.195
NMSS olfaction	0.010	0.007	0.105	0.078	−0.051	0.668	0.105	0.033
NMSS pain	0.092	0.002	0.719	0.195	−0.035	0.100	0.027	0.162
NMSS sexual	−0.045	0.140	0.006	0.253	0.019	0.220	0.775	0.116
NMSS urinary	0.321	−0.022	0.321	0.004	0.106	0.125	0.667	−0.121
NMSS gastro−intestinal	0.396	0.025	0.597	−0.130	0.158	0.172	0.067	−0.043
NMSS attention/memory	0.207	0.634	0.433	0.230	0.059	0.228	−0.064	0.054
NMSS hallucinations	0.190	0.767	−0.123	0.130	0.083	−0.062	0.127	−0.131
NMSS affect/cognition	0.056	0.158	0.400	0.742	0.231	0.018	0.108	0.027
NMSS sleep/fatigue	0.062	0.368	0.440	0.093	0.514	0.028	0.261	0.131
NMSS cardiovascular	0.043	0.071	0.619	0.139	0.154	0.102	0.095	−0.089

Values are correlation coefficients (after varimax rotation).

**Table 3:** Influence of MS and NMS symptoms (factors) on HRQL and ADL.

	EQ-Index			EQ-VAS			Schwab and England			UPDRS II		
	Stan β	B (95% CI)	p value	Stan β	B (95% CI)	p value	Stan β	B (95% CI)	p value	Stan β	B (95% CI)	p value
Factor 1 (motor dysf.)	−0.372	−0.093 (−0.123;−0.063)	<0.0001	−0.296	−6.517 (−9.898; −3.135)	0.0002	−0.672	−10.543 (−12.277;−8.810)	<0.0001	0.696	5.575 (4.850;6.300)	<0.0001
Factor 2 (sleepiness/psychosis)	−0.026	−0.007 (−0.037;0.024)	0.668	−0.188	−4.703 (−8.603−0.802)	0.019	−0.309	−0.309 (−6.590;−3.123)	<0.0001	0.366	2.933 (2.208;3.659)	<0.0001
Factor 3 (cardio/gastro/pain)	−0.358	−0.089 (−0.120;−0.059)	<0.0001	−0.158	−3.427 (− 6.737;−0.118)	0.043	−0.215	−3.370 (−5.104;−1.636)	0.0001	0.283	2.268 (1.543;2.993)	<0.0001
Factor 4 (dep/apathy)	−0.434	−0.109 (−0.139;−0.078)	<0.0001	−0.375	−8.123 (−11.407;−4.839)	<0.0001	−0.167	−2.621 (−4.355;−0.888)	0.003	0.140	1.125 (0.399;1.850)	0.003
Factor 5 (insomnia/anxiety)	−0.347	−0.087 (−0.117;−0.057	<0.0001	−0.260	−5.655 (−8.938;−2,372)	0.001	−0.097	−1.516 (−3.250;−0.218)	0.086	0.150	1.202 (0.477;1.928)	0.001
Factor 6 (olfaction/weight/hyperhidrosis)	−0.016	−0.004 (−0.034;0.026)	0.793	0.082	1.760 (−1.479;4.998)	0.284	0.013	0.197 (−1.536; 1.931)	0.822	0.018	0.144 (−0.581;0.869)	0.695
Factor7 (MoCA/uri./sexual)	0.046	0.012 (−0.019;0.042)	0.446	0.135	2.943 (−0.384; 6.270)	0.082	−0.103	−1.616 (−3.349; 0.118)	0.067	0.134	1.075 (0.350;1.801)	0.004
Factor 8 (Tremor/RBD)	0.015	0.004 (−0.026;0.034)	0.805	0.023	0.493 (−2.838;3.824)	0.770	0.070	1.105 (−0.628; 2.839)	0.209	0.397	0.397 (−0.328;1.122)	0.281

Multiple Linear Regression Analysis results: independent variables are the factors reached by principal component analysis; the Dependent variables are HRQL scale scores (EQ-Index, and EQ-VAS) and ADL scale Scores (Schwab and England and UPDRS part II).
